# A Systematic Review of What Barriers and Facilitators Prevent and Enable Physical Healthcare Services Access for Autistic Adults

**DOI:** 10.1007/s10803-019-04049-2

**Published:** 2019-05-23

**Authors:** David Mason, Barry Ingham, Anna Urbanowicz, Cos Michael, Heather Birtles, Marc Woodbury-Smith, Toni Brown, Ian James, Clare Scarlett, Christina Nicolaidis, Jeremy R. Parr

**Affiliations:** 1 Institute of Neuroscience, Newcastle University, Sir James Spence Institute level 3, Royal Victoria Infirmary, Newcastle upon Tyne, NE1 4LP UK; 2 Northumberland, Tyne & Wear NHS Foundation Trust, Newcastle upon Tyne, UK; 3 Global, Urban and Social Studies, RMIT University, Melbourne, VIC Australia; 4 London, UK; 5 Newcastle University, Newcastle upon Tyne, UK; 6 Campus for Ageing and Vitality, Newcastle General Hospital, Newcastle upon Tyne, UK; 7 NHS North Tyneside CCG, North Shields, UK; 8 Portland State University, Portland, USA

**Keywords:** Autism, Adult, Healthcare access, Physical health, Barriers, Sensory sensitivities

## Abstract

Autistic people are more likely to: be diagnosed with a range of physical health conditions (i.e. cardio-vascular disease); experience premature mortality (for most disease categories); and experience barriers to effectively accessing healthcare. This systematic review sought to identify studies that report on barriers and facilitators to physical healthcare access for autistic people. A total of 3111 records were screened and six studies were included: two quantitative, two qualitative, and two mixed-methodology studies. Patient-provider communication, sensory sensitivities, and executive functioning/planning issues emerged as important barriers to healthcare. Recommendations for clinicians and those planning services are discussed.

**Electronic supplementary material:**

The online version of this article (10.1007/s10803-019-04049-2) contains supplementary material, which is available to authorized users.

There is a growing research literature regarding autistic adults, and their life experiences. One focus has been a range of health and healthcare challenges for autistic adults. Autism is associated with a wide range of co-occurring mental health conditions such as depression and anxiety (Lever and Geurts 2016). Whilst rates of diagnoses and general population comparisons are difficult due to measurement and sampling issues (Howlin and Magiati 2017), autistic people are more likely than the general population to experience elevated rates of co-occurring mental health conditions (Croen et al. 2015; Howlin and Magiati 2017). In addition, there is an elevated rate of co-occurring physical health conditions compared to the general population (Cashin et al. 2018). For instance, data suggest that autistic adults compared to non-autistic people, are more likely to be diagnosed with epilepsy, cardiovascular disease (i.e. dyslipidaemia, hypertension,), and diabetes (Croen et al. 2015).

There is an elevated risk of premature mortality for autistic people compared to the general population. Using two nationwide population-based Swedish registers Hirvikoski et al. (2016) reported significantly elevated rates of mortality for autistic people compared to the general population (Hirvikoski et al. 2016). Further analysis of cause-specific mortality revealed that for all recorded categories of disease, except infection, autistic people were at a greater risk of mortality compared to the general population (i.e. OR = 3.7 for endocrine, OR = 1.5 for circulatory system, or OR = 3.3 for digestive system diseases). These findings were similar to rates in Denmark, with comparable sample sizes (Schendel et al. 2016).

Regarding service use, autistic people are more likely to access some healthcare services compared to the general population. Vohra et al. (2016) reported autistic people made significantly more emergency department visits (for both physical and mental health reasons) compared to the general population (although fewer visits for alcohol and substance misuse disorders, and respiratory disease) (Vohra et al. 2016). Results from a recent study comparing health records for 1507 autistic people and 15,070 members of the general population indicated autistic people were 2.1 times more likely to attend an outpatient healthcare visit (Zerbo et al. 2018). A separate study found that younger autistic adults were more likely to visit a general practitioner (OR = 1.27) or be hospitalised (OR = 2.75) compared to age-matched general population controls (Weiss et al. 2018). However, autistic adults are significantly more likely to report unmet healthcare needs, and lower healthcare self-efficacy, compared to the general population (Nicolaidis et al. 2013) and there remain variabilities in which services are accessed: For instance, autistic people may be more likely than the general population to access a screening service for diabetes but not prostate, cervical, or breast cancer screening (Zerbo et al. 2018). This elevated use of services may reflect autistic people trying to find a healthcare provider with a good level of knowledge about autism (Unigwe et al. 2017; Zerbo et al. 2015, 2018).

Many factors potentially relate to effective health care for autistic people. Survey and interview data show many healthcare providers do not have sufficient skills or tools to effectively treat autistic people (Zerbo et al. 2015); providers may not receive formal autism related training (Unigwe et al. 2017). A large survey recently found autistic people reported significantly lower satisfaction with patient-provider communication, and healthcare self-efficacy, and had significantly more unmet needs compared to the general population (Nicolaidis et al. 2013). Communication is a two way process, and whilst social communication is a core difficulty for autistic people, professionals may not appreciate the need to adapt their communication style to communicate effectively with autistic people—or be unable/unwilling to do so. For example, some healthcare providers are not amenable to the use of augmented communication; assuming the autistic patient cannot attend the appointment without a relative or carer (Nicolaidis et al. 2015).

Taken together, the research literature suggests autistic people have an increased prevalence of some health conditions; those contribute in part to the increased rates of premature mortality; and there are numerous barriers preventing autistic people from effectively engaging with the appropriate healthcare practitioners to increase the likelihood of their health needs being met. Recently a UK community priority setting exercise identified ‘How should service delivery for autistic people be improved and adapted in order to meet their needs?’ as a research priority (James Lind Alliance 2016). In that context, understanding the barriers to healthcare access is key to implementing changes to services.

The objective of this review was to systematically review and synthesise the current research into the barriers and facilitators to healthcare access experienced by autistic people. The specific question addressed by this review was: ‘what barriers and facilitators prevent and enable physical healthcare services access for autistic adults?’

## Review Methods

The Preferred Reporting Items for Systematic Reviews and Meta-Analyses (PRISMA) guidelines were used throughout the review (Moher et al. 2009), which was registered on PROSPERO (the international prospective register of systematic reviews: www.crd.york.ac.uk/prospero/). ID CRD42018110516.

### Review Criteria

Peer-reviewed quantitative (i.e. randomised control trials, observational studies), qualitative (i.e. interview or focus group) studies, and mixed methodology studies, published in English from any country of origin were eligible for inclusion in the review. The inclusion criteria were: Studies including or relating to autistic adults aged 16 years and over (primary sample/discrete sub-sample), or studies including a quantitative or qualitative description of barriers to physical healthcare access, or characteristics that promoted access to physical healthcare for autistic people. Studies that investigated which services autistic people access/are more likely to access were not eligible for inclusion. To decide whether a paper should be included, the first author, and HB, used a decision matrix. This matrix consisted of key questions to address the inclusion/exclusion criteria consistently (see Supplementary material Table S1).

### Search Strategy

The authors designed and piloted the initial search strategy and search terms and consulted relevant experts to refine them further. The final list of search terms contained key words taken from the literature and MeSH (Medical Subject Headings) terms.

The first author searched the following databases: CINAHL, Web of Science, MEDLINE, Embase, and PsychINFO. Searches were limited to post 1994 to cover the DSM IV and DSM-5 eras. Key search terms, in different permutations, were: autis*, Asperge*, developmental dis*, barrier, facilitator, booster, adjustment, accommodation, access*, utili*, healthcare, delivery of care, delivery of healthcare, health checks, health services, general practitioner, physician, primary healthcare. In parallel, experts in this area were contacted, and a search of Google Scholar and relevant policy and health reports was conducted to identify additional citations.

The initial search, removal of duplicates, and full text read-throughs was completed by 31st October 2018. All searches were rerun from the 1st November 2018 up to the 24th of January 2019. No additional papers were included.

### Study Selection Process

After excluding duplicates, the first author screened articles by title and abstract against the inclusion criteria. A second reviewer, HB, checked a randomly selected 10% of the abstracts against the inclusion criteria to investigate agreement (99.6%). The first author then completed a full text read through of each study against the inclusion criteria. Several articles were thought to partially meet the inclusion criteria; the final decision was decided by group consensus by all authors.

Data extraction for the final sample of studies was completed using a data collection form to record study sample size, population, design, any intervention(s) and outcome measures used, and the main findings. If further information was required, corresponding authors were contacted for clarification.

### Assessment of Methodological Quality

Studies included in the final data synthesis were evaluated for methodological quality. This was conducted independently by two reviewers (two-way intraclass correlation coefficient = 0.97, *p *< 0.001 indicated excellent agreement of ratings). For all studies the methodological reliability and validity was evaluated using the Quality Assessment Tool for Studies with Diverse Designs (QATSDD) (Sirriyeh et al. 2012). This is a 16-item tool with 14 items relevant to quantitative or qualitative studies (all 16 items were scored for mixed-methodology studies). Each item is scored from 0 to 3, thus quantitative or qualitative scores can range from 0 to 42, or 0–48 for mixed-methodology studies. This tool was selected as, a priori, it was expected that included studies would use a range of methodologies, and the QATSDD offers a numerical score for comparisons.

### Data Synthesis

We chose a narrative approach to data synthesis because we expected, a priori, to find few quantitative studies and that any quantitative studies would include highly heterogeneous operationalisation of ‘barriers’. Furthermore, it was expected that qualitative synthesis would represent the qualitative research most effectively.

## Results

### Study Selection

**Fig. 1:**
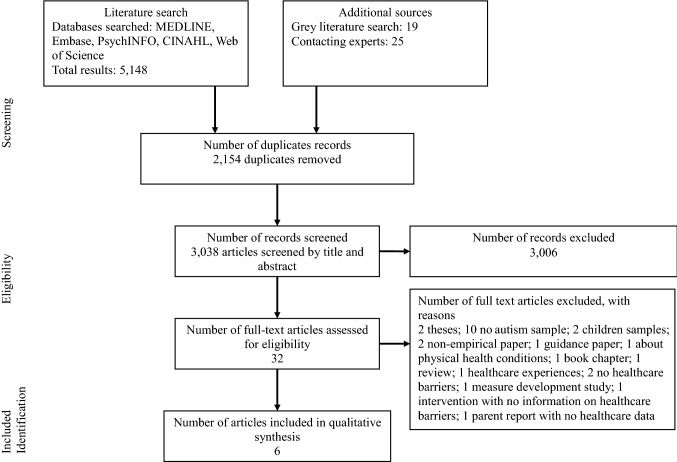
PRISMA diagram showing the process used to identify studies, study search and selection

The search strategy yielded a total of 5192 records. After removing duplicates, a total of 3038 records were screened by title and abstract. A total of 3006 records were excluded at this stage. This left 32 studies for a full text review. Post review, six studies were included. The most common reason for exclusion was a lack of autistic participants. This is because the search strategy allowed for the inclusion of studies that included people with intellectual disability (who may or may not have included sub-samples of autistic participants). None of the studies describing intellectual disability participants met the inclusion criteria as they did not report the number of autistic people, or present the findings from autistic people. See Fig. 1 for a flowchart of the search process, and list of reasons for exclusions.

### Study Characteristics

Of the six papers selected, two were qualitative (Dern and Sappok 2016; Nicolaidis et al. 2015), two were quantitative (Raymaker et al. 2017; Vogan et al. 2017), and two used mixed-methods (Nicolaidis et al. 2016; Saqr et al. 2018). Sample sizes ranged from 10 (Saqr et al.’s focus group; Saqr et al. 2018) to 209 (Raymaker et al. 2017). The combined sample size of autistic adults was 683; three studies (Nicolaidis et al. 2015, 2016; Raymaker et al. 2017) included 229 non-autistic supporters, clinicians, adults with disabilities, and adults without disabilities. One study did not provide sample size data (Dern and Sappok 2016). Table 1 describes the included studies.

**Table 1:** Description of the focus, method and main findings from the included studies

Study	Population	Focus	Research type and study design	Findings
Nicolaidis et al. (2015)	39 autistic adults (who report an autism spectrum diagnosis); mean age 35 years (19–64) 16 supporters of an autistic person(s); mean age 52 years (28–74)	To obtain an in-depth understanding of autistic adults’ experiences with healthcare and their recommendations for improving care	*Qualitative*. Individual interviews (participants could respond to questions via telephone, e-mail, or instant messenger); thematic analysis with an inductive approach at a semantic level	Identifies three clusters of barriers: Patient-level factors (e.g. verbal communication skills, slow processing speed); provider-level factors (e.g. knowledge about autism in adults, use of accessible language); and system-level factors (e.g.. availability of formal/informal support, stigma about autism)
Dern and Sappok (2016)	Autistic self-advocates and autism professionals (sample size and composition are not described)	The available experiences of autism self-advocates and clinical experiences of practitioners	*Qualitative*. Summation of key meetings from a 5 year (2006–2011) series of meetings between autism self-advocates and healthcare professionals	Identifies barriers to healthcare for autistic people (i.e. difficulties making phone calls, lack of time to think/respond or use written notes). Recommends how professionals can improves healthcare for autistic adults (i.e. alternative methods to make appointments, allow patient to make notes/record discussions)
Nicolaidis et al. (2016)	259 autistic adults who took part in cognitive interviews (n = 30, mean age 37.6 years, 20–64), test–retest reliability (n = 59, mean age 34.6, 18–64), pre- and post-intervention surveys (n = 170, mean age 36.5, 18-68); 51 primary care providers who took part in cognitive interviews (n = 10, mean age 41.6, 27–61), and a post-intervention survey (n = 41, mean age 36.3, 28–62)	Using community-based participatory research to create and evaluate an online healthcare toolkit for autistic adults and their primary care providers	*Mixed methodology.* Cognitive interviewing and test–retest studies. Toolkit evaluation was a single arm pre-/post-test intervention comparison of surveys with closed- and open-ended items	Almost all autistic participants and supporters rated the Toolkit as easy to use, important, and useful Most primary care providers rated the Toolkit as moderately or very useful and indicated they would recommend it to their patients Over the course of the intervention the number of self-reported barriers to healthcare reduced significantly from a mean of 4.1 to 2.8 (with healthcare self-efficacy scores also increasing significantly from 37.9 to 39.4 and satisfaction with patient-provider communication scores increasing significantly from 30.9 to 32.6)
Raymaker et al. (2017)	209 autistic adults (mean age 37 years, SD = 13); 55 adults in a disability group (mean age 45, SD = 14); 174 adults in a non-autistic non-disabled group (mean age 38, SD = 12)	Identify and compare barriers to accessing healthcare experienced by autistic adults and adults with, and without other disabilities	*Quantitative*. Cross-sectional instrument development and validation (Long- and Short-Form Barriers to Healthcare Checklist)	Autistic adults and adults with other disabilities endorsed significantly more barriers than the non-autistic adults without disabilities. Autistic adults selected a different pattern and a greater number of barriers that adults with other disabilities, particularly in areas related to emotional regulation, patient-provider communication, sensory sensitivity, and healthcare navigation
Vogan et al. (2017)	40 autistic adults (mean age 35.9, SD = 11.7)	Autistic adults access of healthcare services, experiences of accessing healthcare services, barriers to service use, and reported unmet service needs	*Quantitative*. Longitudinal study over a 12-18 month time frame, with participants completing measures every two months	The most commonly reported barriers by participants were not knowing where to find help (65.8%), overwhelming steps to seek help (52.6%), and negative experiences with professionals (47.4%). Over 75% endorsed three or more barriers to healthcare. Those with medical problems reported significantly more barriers to healthcare than those without (5.12, SD = 2.65; 3.20, SD = 2.25 respectively)
Saqr et al. (2018)	126 autistic adults who took part in a retrospective chart analysis (mean age 21.2, SD = 5.6); 10 autistic adults who took part in a focus group (ages 18–30)	Environmental and process barriers to care access in a primary care setting and to examine medication use in the sample	*Mixed methodology*. Cross-sectional retrospective chart analysis; focus group	74 individualised plans were created for patients. The most common adjustment was taking the patient to the examination room upon arrival and completing registration there (n = 16) Focus group data highlighted the most stressful parts of the healthcare visit: waiting (both in the waiting room and examination room, and the examination). Furthermore a negative feedback loop (fear of social interaction, heightens anxiety and overstimulation which makes social interaction difficult) was identified

### Results of Individual Studies

Qualitative studies: Two qualitative studies (Dern and Sappok 2016; Nicolaidis et al. 2015) investigated the experiences of autistic adults when accessing healthcare. Nicolaidis et al. (2015) also recruited a sample of supporters of autistic adults as they may be the main communicators with healthcare professionals when the person they support accesses healthcare. Dern and Sappok (2016) collected data from an existing collaboration between autistic self-advocates and healthcare professionals treating autistic people. Both studies identified an extensive list of barriers that impair or prevent autistic people from accessing health care, and recommendations for improving healthcare provision.

Each study identified a set of barriers and a unique taxonomy of these barriers. Nicolaidis et al. (2015) identified patient-level factors (i.e. verbal communication skills, sensory sensitivities, body awareness, slow processing speed, atypical non-verbal communication, and challenges with organization); provider-level factors (i.e. knowledge about autism in adults, incorrect assumptions, flexibility in following the patient’s preferred communication style, use of acceptable language, openness to proving accommodations, and skills in incorporating supporters); and systemic-level factors (i.e. available formal or informal supports, complexities and accessibility of the healthcare service, and stigma about autism). In comparison, Dern and Sappok (2016) identified five sets of barriers: making appointments (i.e. telephone calls can be challenging); waiting area (i.e. uncertain wait times, proximity of other patients, overly-stimulating noise level/lighting); examination (i.e. discomfort with unannounced touch, atypical presentation and experience of pain); communication (i.e. open-ended questions causing stress, patient cannot use written notes, idiosyncratic or hyper-specific language); hospital (i.e. constantly changing staff, not being admitted due to rigid thinking about what is happening at home); and sensory difficulties (i.e. prosopagnosia, sensory overload through traveling to, or being at, appointments).

Quantitative: One quantitative study tracked the healthcare use, satisfaction with healthcare, and barriers to accessing healthcare by a sample of autistic adults (Vogan et al. 2017). A second study described the development and validation of a measure of barriers to accessing healthcare (Raymaker et al. 2017). Each study addressed a different research question yet common barriers to healthcare were identified.

Raymaker et al. (2017) adapted an existing measure of barriers to healthcare used with disability populations. Using a community-based participatory research approach, the research team modified items for accessibility and added autism-specific barriers identified in previous research (Nicolaidis et al. 2013, 2015). The final version of the measure showed acceptable content and construct validity (as established by the research team and the pattern of item endorsement respectively). The long-form of the measure consists of 41 items across nine domains: emotional (i.e. frustration/anger, lack of confidence); executive function (i.e. organising appointments, translating medical instruction in practicable steps); healthcare navigation (i.e. find it too hard to navigate managed care, problems with paperwork); provider attitudes (i.e. providers relate new symptoms to existing conditions, providers are unwilling to use the patient’s preferred communication style); patient-provider communication (i.e. difficulty communicating with providers, trouble following spoken instructions); sensory (i.e. healthcare facilities cause sensory overload); socio-economic (i.e. concerns about costs, insurance does not cover atypical therapies); support (i.e. socially isolated, lack of available support); and waiting (i.e. waiting rooms are difficult to manage). The short form of the measure consists of 17 items. The short-form items cover the same nine domains as the long-form measure; items were created by collapsing conceptually similar items into one item and removing more general items in favour of more specific items (Raymaker et al. 2017).

Vogan et al. (2017) reported similar barriers, e.g. not knowing where to find help, too many steps to finding help, and negative experiences with healthcare staff. Furthermore, Vogan and colleagues noted that around three quarters of participants could not access healthcare they needed, and over three quarters reported three or more barriers to healthcare access. Moreover, those experiencing physical health conditions reported almost twice the amount of barriers to healthcare than those without medical conditions.

Mixed-methods: Two studies (Nicolaidis et al. 2016; Saqr et al. 2018) sought to measure barriers to healthcare access, augmenting survey data with descriptive accounts (interviews and focus groups) from autistic people and, for Nicolaidis et al. (2016), primary care providers. Saqr et al. (2018) conducted a retrospective medical record review of patients attending a primary care facility set up to facilitate transition for autistic people (Saqr et al. 2018); Nicolaidis et al. conducted a single arm pre-/post-intervention evaluation of an online toolkit, the Autism Healthcare Accommodations Toolkit (AHAT) using surveys of autistic patients and their providers (Nicolaidis et al. 2016). Saqr et al. collected qualitative data about the clinical experience of autistic people and Nicolaidis et al. also used cognitive interviews to assess the content validity of the AHAT.

One study, Saqr et al. (2018) used a standardised pre-visit assessment to identify possible barriers to a successful healthcare visit. An individualised plan was then made for patients who may experience such barriers. Saqr et al. reported that 23% of the patients in their sample (n = 17) had some form of individual plan. Qualitative data identified three sets of problems that interact with the healthcare visit. Participants suggested that *sensory sensitivities* made check-in and the waiting room a stressful experience. *Anxiety from waiting* made the waiting in the examination room and talking with the healthcare provider challenging. Participants suggested that *lack of mutual understanding, communication, and trust* made both the examination and treatment conversation more stressful. The authors then proposed that a negative feedback loop of social interaction developed during the healthcare visit. In brief, fear of social interaction drives anxiety and stress, which in turn heightens sensory sensitivity and overstimulation. This impacts concentration and makes social interaction more difficult, consequently increasing fear of social interaction. Nicolaidis et al. designed the AHAT to be completed by an autistic person or their supporter. The items were generated from previous research into healthcare barriers (Nicolaidis et al. 2013, 2015). The patient completes the sections of the AHAT: How you communicate; Communication suggestions; Before the visit; During the visit; After the visit; Getting to know you; and Your supporters. The AHAT then generates an individualised report than can be shared with the primary care provider. From the cognitive interviews, both autistic people and primary care providers responded positively to the AHAT, rating it as helpful and stating it included a comprehensive list of accommodations. Autistic people reported feeling more validated in their experiences and more able to self-advocate. Primary care providers said they would recommend it to other autistic patients. The AHAT significantly reduced healthcare barriers over the course of the evaluation intervention, as measured by the Barriers to Healthcare Checklist—Short Form, and increased satisfaction with patient-provider communication. Open-ended questions suggested that the toolkit empowered patients’ self-efficacy and that patients noted some changes in primary care providers’ behaviour. Whilst some patients reported that their primary care provider did not read the AHAT or did not change their practice after receiving it, most primary care providers in the evaluation rated the AHAT as moderately or very useful.

### Synthesis of Results

Research investigating barriers to healthcare for autistic adults to date has used a diverse range of methodologies and measures. Qualitative studies have been used to elicit the lived experiences of autistic adults when accessing healthcare, providing recommendations for improving healthcare, and to posit tentative models of the healthcare visit. Furthermore, qualitative data has been integrated into validating measures of healthcare barriers. All studies included samples of autistic adults (aggregate age range approximately 18-64). One study compared autistic adults to the general population and to non-autistic people with other disabilities groups (Raymaker et al. 2017); two studies only included autistic participants (Saqr et al. 2018; Vogan et al. 2017); three studies included groups of supporters (Nicolaidis et al. 2015) or healthcare professionals (Dern and Sappok 2016; Nicolaidis et al. 2016).

Despite diverse measures of barriers (for quantitative studies) and interview guides (qualitative studies), a consistent set of obstacles emerged across studies (see Table 2). Communication with healthcare providers was reported across five studies as a barrier to healthcare (Dern and Sappok 2016; Nicolaidis et al. 2015, 2016; Raymaker et al. 2017; Saqr et al. 2018). Communication difficulties included: checking into a healthcare visit (Saqr et al. 2018), communicating with the primary care provider (Dern and Sappok 2016; Nicolaidis et al. 2015; Saqr et al. 2018), or providers not respecting the autistic person’s preferred communication method (Dern and Sappok 2016; Nicolaidis et al. 2015). Negative experiences with healthcare providers was also a prominent barrier (Nicolaidis et al. 2015). Vogan et al. found that 47.4% of their sample reported such experiences (Vogan et al. 2017).

**Table 2:** Barriers to healthcare access reported across studies (listed in order of consistency of findings across studies)

Barrier(s)	Studies					
	Nicolaidis et al. (2015)	Dern and Sappok (2016)	Nicolaidis et al. (2016)	Raymaker et al. (2017)	Vogan et al. (2017)	Saqr et al. (2018)
Communication (i.e. atypical communication, literal interpretation, making appointments)	+	+	+	+		+
Sensory Sensitivities (including the waiting room, physical examination)	+	+	+	+		+
Challenges with bodily awareness (i.e. difficulty describing pain or symptoms)	+	+	+	+	+	
Providers’ degree of flexibility (i.e. allowing written communication, using accessible language, making needed accommodations)	+	+	+	+		+
Slow processing speed (i.e. during social interaction) or executive functioning (i.e. self-regulating medication, missing appointments)	+	+	+	+		
Providers’ negative attitudes (i.e. misinterpreting behaviours, communication is not taken seriously)	+		+	+	+	
Availability of supports (both formal and informal; fear of social isolation)	+		+	+	+	
Healthcare system is too complex or inaccessible (including not knowing where to find help)	+		+	+	+	
Emotional (i.e. anxiety or embarrassment)*			+	+	+	+
Challenges with organisation (i.e. remembering to take medication, making or attend appointments)	+	+	+	+		
Need for consistency (i.e. seeing the same staff)	+	+	+			
Providers’ (lack of) knowledge about autism in adults (including making assumptions about behaviour, or lacking confidence in treating autistic patients)	+		+	+		
Negative experiences with healthcare (including lack of trust in professional help, not including the autistic patient in healthcare discussions)	+			+	+	
Stigma about autism	+		+		+	
Other societal issues that affect health (including socio-economic factors)	+			+		
Highly variable needs of autistic people			+			+
Distance too far to get help				+	+	
The problem did not seem so serious					+	
Want to handle the problem ourselves [the autistic person]					+	
Too busy/other priorities					+	
Problem was considered temporary					+	
Other people did not want the family to seek help					+	

+ Barrier is reported by this study

Nicolaidis et al.’s qualitative study provided rich data regarding barriers to healthcare. This study reported that healthcare providers’ knowledge about autism can act as a barrier to healthcare. For instance, qualitative reports from autistic adults suggest that many healthcare providers do not know what autism entails and hence how treatment may need to be adapted (Nicolaidis et al. 2015). One key finding, relevant to clinicians, is that assumptions made by healthcare providers based on the presentation of the autistic patient (Nicolaidis et al. 2015). For instance, if the autistic patient requests that healthcare providers use an alternative communication device then they may assume the patient lacks the ability to comprehend what is said. Alternative communication preferences have been reported in several studies. For instance, written notes may be preferred due to challenges with verbal and non-verbal communication (Dern and Sappok 2016).

Sensory sensitivities were a prominent barrier across five studies (Dern and Sappok 2016; Nicolaidis et al. 2015, 2016; Raymaker et al. 2017; Saqr et al. 2018). Different aspects of the healthcare visit are associated with sensory sensitivities. For instance, the waiting room (Dern and Sappok 2016; Nicolaidis et al. 2016; Saqr et al. 2018), unpredictable waiting times (Nicolaidis et al. 2016; Saqr et al. 2018), travelling to appointments (Dern and Sappok 2016; Saqr et al. 2018), lighting and environmental factors (Nicolaidis et al. 2015; Saqr et al. 2018) can all contribute to sensory overload. Interestingly, Saqr et al.’s qualitative data suggest that the stress and anxiety about a healthcare visit begins prior to the visit (i.e. at home, or when travelling to the healthcare venue) (Saqr et al. 2018).

Finally, a set of intra-person factors were reported across several studies as barriers to accessing healthcare. These included a slower processing speed when talking with providers (Nicolaidis et al. 2015) which could hamper ‘real-time’ interactions during an appointment (Raymaker et al. 2017). Less often mentioned, but important none the less, were issues of information processing and memory. Raymaker et al.’s Barriers to Healthcare Checklist included a domain about executive functioning (i.e. remembering to attend appointments or take medication) and difficulties with completing paper work were noted (Nicolaidis et al. 2015; Raymaker et al. 2017).

### Methodological Quality of Included Studies

Many studies relied on self-reported diagnoses of being on the autism spectrum. For instance, Nicolaidis et al. (2015, 2016) required that participants report a formal or medical diagnosis of autism, Asperger’s syndrome, pervasive developmental disorder not otherwise specified, or autism spectrum disorder. Nicolaidis et al. (2015) also allocated a portion of their sample to those who self-identified as being on the autism spectrum, with the autism quotient (AQ; Baron-Cohen et al. 2001) used as a screen to indicate autistic traits. Raymaker et al. (2017) likewise recruited participants who identified as being on the autism spectrum (Raymaker et al. 2017). For their retrospective chart analysis Saqr et al. (2018) identified all patients who attended a specific healthcare provider between April 2014 and April 2015, and autism diagnosis was confirmed by a review of medical records and psychological evaluation (Saqr et al. 2018). Finally one study did not provide any participant information (Dern and Sappok 2016).

Quantitative measures used in the included studies were highly heterogeneous, and in some cases not validated for use with this population (or authors did not comment on the measures’ validities). Four studies used quantitative data collection methods in their designs. Two of these studies sought to validate a measure or tool for use with autistic adults accessing healthcare (Nicolaidis et al. 2016; Raymaker et al. 2017). The initial validation findings were promising, suggesting that each tool is appropriate for use with autistic participants in future studies or interventions. One study looked at retrospective chart analyses for every patient attending a specific healthcare provider over a 12 month period (Saqr et al. 2018). The complexity of each patient’s medication regimen was analysed. The authors do state the measure of complexity is valid for use with chronic disease, but the measure of medication regimen complexity has not yet been validated for use with autistic people. Finally, Vogan et al. used several measures including the Kessler Psychological Distress Scale-6 and the Need for Help Questionnaire (Vogan et al. 2017). One measure of service use was created by the research team (‘service use’). Neither the Kessler Psychological Distress Scale-6 nor the Need for Help Questionnaire have been validated for the autistic population.

## Discussion

We identified six papers investigating the barriers and facilitators to healthcare as reported by autistic adults. Despite the limited literature, there are similar barriers and facilitators reported across the six studies; efforts to devise interventions and measurement tools are underway, and initial steps towards evaluating the effectiveness of implementing change have been evidenced (Nicolaidis et al. 2016; Dern and Sappok 2016; Saqr et al. 2018; Vogan et al. 2017; Nicolaidis et al. 2015; Raymaker et al. 2017).

Nicolaidis et al. reported that verbal communication skills were an autism-related factor affecting healthcare access (Nicolaidis et al. 2015). Social or communicative atypicalities are part of the diagnostic criteria for autism spectrum conditions (McPartland et al. 2012), however, autistic people display highly heterogeneous profiles of social and communicative ability (Frazier et al. 2012). Thus, social communication accommodations required by autistic patients are likely to be highly heterogeneous and unique to each patient. General difficulties in verbal and non-verbal communication can act as a barrier to healthcare, or a lack of initiative in reporting medical conditions (Dern and Sappok 2016). By way of example, literal interpretation of language was reported to impair some autistic people’s ability to answer questions about, for instance, quantifying pain (Nicolaidis et al. 2015). These findings suggest that healthcare practitioners should be sensitive to the communication atypicalities associated with autism spectrum conditions. A ‘one size fits all’ approach to patient interaction is unlikely to work for autistic people; hence efforts to create individualised reports tailored to the autistic patient’s needs (Nicolaidis et al. 2016; Hislop et al. 2016). Given autistic patients are more likely to report barriers to healthcare compared to both adults with disabilities (vision/hearing, mobility, learning/remembering, activities of daily living, leaving home alone, working at a job) and non-autistic adults without disabilities (Raymaker et al. 2017), such attention to individual needs may reduce barriers to healthcare (Nicolaidis et al. 2016). For example, negative experiences with healthcare providers (Vogan et al. 2017) and ‘adverse events’ that might affect medical procedures (i.e. measuring vital signs) or increase anxiety about attending healthcare appointments (Saqr et al. 2018).

Importantly, these communication difficulties only represent one aspect of barriers to healthcare. Several studies reported different barriers related to stigma or stereotyping, healthcare providers’ lack of autism knowledge, healthcare providers’ openness to different modes of communication, and trust in professionals. Almost one quarter of autistic people in Vogan et al.’s study reported a fear of stigmatisation or labelling (Vogan et al. 2017); although the sample size was small (N = 40) this does echo the findings of other studies that used qualitative methods. For instance, some autistic people who use alternative communication devices reported that their healthcare professional doubted the autistic patient’s competence (Nicolaidis et al. 2015). Provider attitudes towards autistic patients was discussed in qualitative studies (Dern and Sappok 2016; Nicolaidis et al. 2015) and was a discrete factor in the ‘Barriers to Healthcare Checklist- Long Form’ (Raymaker et al. 2017). For instance, healthcare providers were reported to make assumptions about autistic patients from their initial presentation (Nicolaidis et al. 2015), not take into account rigid thinking (Dern and Sappok 2016), or assume behaviours expressing symptoms (i.e. pain) were behaviours more directly related to autism (Dern and Sappok 2016). These findings are in line with healthcare professionals’ self-reporting. For instance, 79% of adult medicine, 88% of obstetrics/gynaecology, and 70% of mental health professionals in the United States rated their ability to provide healthcare to autistic patients as poor or fair (Zerbo et al. 2015). Furthermore, in the United Kingdom 39.5% of general practitioners (n = 120 out of 304) reported having no formal training in autism, and limited confidence in their ability to offer healthcare to autistic patients (despite having a good knowledge of the key features of autism) (Unigwe et al. 2017). Again, these findings have important implications for healthcare professionals. It is important that healthcare professionals with autistic patients take the time to learn about autism spectrum conditions, and the individual needs of autistic patients. Nicolaidis et al. reported that the AHAT was rated positively by primary care providers (82% rated it as moderately or very useful) (Nicolaidis et al. 2016).

The final ‘global’ barrier reported was sensory sensitivities. Autistic people report elevated sensory processing sensitivity compared to the general population (Crane et al. 2009; Tavassoli et al. 2014). This over-sensitivity is across a range of sensory modalities (sight, taste, touch, sound, smell) and can therefore have a significant impact on daily living (Tavassoli et al. 2014). The studies in this review agree with these findings. Compared to both non-autistic adults without disability (vision/hearing, mobility, learning/remembering, activities of daily living, leaving home alone, working at a job) and adults with disability, autistic people endorsed significantly more sensory items (e.g., lights/smells/sounds make visits uncomfortable or communicate well in healthcare settings) (Raymaker et al. 2017). Lighting (too harsh or bright), crowded waiting rooms (noise, smell, proximity of others), and unpredictable waiting times were also reported to be sources of sensory overload (Nicolaidis et al. 2015). Saqr et al. describe a ‘feedback’ model in which sensory overload reinforces feelings of anxiety, exacerbating communication difficulties when accessing healthcare (Saqr et al. 2018). These findings are important for ensuring the healthcare visit goes well. Healthcare settings should seek to minimise sensory sensitivities where possible. This could include having quiet waiting areas for autistic patients or allowing autistic patients to wait outside the healthcare building and being brought directly into their healthcare appointment.

The intra-person cognitive factors of processing speed, organisation, and memory were not extensively mentioned but have important implications for healthcare settings. The Barriers to Healthcare Checklist includes items about processing speed affecting healthcare discussions and finding it difficult to follow spoken instructions (Raymaker et al. 2017). It is well documented that autistic children struggle to integrate multi-modal sensory information (Marco et al. 2011), or have difficulty processing rapidly presented information (Gepner and Mestre 2002). Evidence suggests that autistic adults are better than the general population at identifying parts of faces, but are poorer at identifying a face holistically (Lahaie et al. 2006). This is important as healthcare appointments with detailed conversations involve processing three streams of information: verbal (listening to the information), visual (looking at the healthcare provider, which some autistic people find aversive, Hurlbutt and Chalmers 2002), and processing (thinking about the information being presented and preparing a verbal response). Thus, an autistic adult may be overwhelmed by the demands of maintaining a social conversation and thinking through the implications of what they are being told. Furthermore, prospective memory (planning ahead for an event, and remembering to carry it out) may be impaired for autistic people. For instance, there are documented problems with timing-based prospective memory (i.e. do event x at time y) in both autistic children (Williams et al. 2013, 2014) and autistic adults (Altgassen et al. 2012). Whilst these are experimental data and may not be entirely valid for healthcare settings, the findings do agree with the anecdotal accounts (Nicolaidis et al. 2015) and measure development (Raymaker et al. 2017) that was identified by this review. Therefore, it is essential that healthcare providers account for these individual differences when interacting with autistic patients. Patients may need more support during healthcare appointments with how health information is disseminated, and more support with making and attending healthcare appointments. The AHAT for primary care providers has information about communication issues for autistic people, and allows the autistic patient to inform their care provider if they have processing speed issues (Nicolaidis et al. 2016). As the AHAT was shown to significantly decrease barriers to healthcare over time (Nicolaidis et al. 2016), accommodating these differences may improve the delivery of healthcare to, and consequently the health of, autistic adults.

Although the age range of participants in the included studies was good (18-64 years) some questions remain about differences in healthcare access needs across the lifespan for autistic people. Currently there is a dearth of research about the characteristics and needs of older autistic adults (Mukaetova-Ladinska et al. 2012; Herrema et al. 2017; Rodgers et al. 2018). Yet, autistic people have many questions about how they will experience the ageing process and the additional support that will be required. For instance, what is the future like for an older autistic person cared for by elderly relatives? (Michael 2016). There is a wealth of literature about the transition experiences of young autistic people (Adreon and Durocher 2007; Hendricks and Wehman 2009; Taylor and Seltzer 2011). Yet, little is known about transitions for older autistic people (Mukaetova-Ladinska et al. 2012; Rodgers et al. 2018). A recent study with autistic adults (mean age 36 years) identified several factors important to autistic people as they contemplate the future (Rodgers et al. 2018). The overarching theme was uncertainty about the future, and specifically, worry about support needs and relationships, living circumstances, and health (Rodgers et al. 2018). These findings agree with a recent survey of 45 individuals who were either autistic individuals or carers of autistic individuals. The survey identified long-term management and awareness of ageing in autism as key research priorities (Mukaetova-Ladinska and Stuart-Hamilton 2015). Taken together, this research highlights the dearth of evidence that could inform healthcare practice for older autistic people. Exploring other literatures could provide a starting point for investigation. For instance, dementia research has looked at promoting a positive hospital experience for patients with cognitive impairment (Prato et al. 2018). Prato and colleagues identified themes that determined the quality of the hospital experience: valuing the person, activities to promote empowerment, and creating a suitable environment to support well-being (Prato et al. 2018).

None of the included studies included samples of autistic people with intellectual disability (ID). It is feasible that some of the excluded studies did in fact have participants with ID who were also on the autism spectrum. However, this review took the position to only include papers that had reported details about samples (or subsamples) of autistic people. Hence, there could be some strategies to improve healthcare access for autistic people with ID that were not included here. For instance, toolkits designed to improve healthcare self-efficacy in ID populations (Lennox et al. 2012) could be tested with both autistic people, and autistic people with co-occurring ID.

A final observation is that overarching healthcare ‘systems’ may impact on healthcare. For instance, Nicolaidis et al. identified system level factors affecting access to healthcare (Nicolaidis et al. 2015). These factors included availability of formal or informal supports and the complexities of the healthcare system (for instance the number of ‘administrative hoops’ patients have to navigate). Indeed, some autistic people reported they felt that they could not navigate the healthcare system without help (Nicolaidis et al. 2015). This is supported by Vogan et al.’s findings whereby just over 50% of participants reported that the steps to seeking help and almost 70% reported that not knowing where to find help were barriers to accessing health (Vogan et al. 2017). These findings do suggest that healthcare professionals could alleviate some difficulties for their autistic patients by providing accurate and concise signposting to healthcare services. Signposting to appropriate services (i.e. towards services the autistic person needs) has been reported to improve access to a range of services (i.e. education and support) and overall well-being in a non-funded community group (Southby and Robinson 2018). This does suggest that directing autistic people to appropriate services does not entail extra costs, and the result could improve *effective* access to care for autistic people.

Overall, the quality of the six included studies was acceptable to good (60–80%, with one study scoring 20%); however, they addressed different questions with an array of methods and some observations can be made. First, the total sample size for each study tended to be small (i.e. number of participants between 40 and 259), and participants from diverse demographic groups were included (or instance, a range of educational attainment and ethnicity, Nicolaidis et al. 2015, 2016; Raymaker et al. 2017); one study did include participants who were from high-income areas, Vogan et al. 2017). Two studies included people with autism self-diagnosis (Nicolaidis et al. 2015; Raymaker et al. 2017); many adults may be autistic, yet lack a formal diagnosis because current adults were less likely to have received a diagnosis in childhood (Stuart-Hamilton and Morgan 2011; Lewis 2016). It is possible that those with a self-diagnosis face comparable barriers to those with a formal diagnosis, however further research is required to determine this. As such, clinicians should be sensitive, or receptive, to those who self-identify as being on the autism spectrum. Second, three studies used a co-designed approach whereby autistic people were involved in choosing research questions, designing the studies, recruitment, consent process, data collection and analysis (Nicolaidis et al. 2015, 2016; Raymaker et al. 2017). Two of these studies evaluated a new tool. One, to help healthcare providers by creating a statement of individualised healthcare adjustments that could help improve the healthcare appointment for the autistic patient (Nicolaidis et al. 2016) and the other a checklist to measure barriers to healthcare access (Raymaker et al. 2017). Both of these tools were reported to have high face validity and adequate measurement properties. It is reasonable to suggest that this co-design process was a key component in the success of developing these measures (also, these three studies obtained the highest ratings in the quality assessment). Future studies could emulate this approach to make research more accessible for autistic people. Third, there is now a validated checklist that healthcare providers and researchers can use to tackle future research questions about healthcare barriers with the autism population (Raymaker et al. 2017). Fourth, there is not yet a consensus on how to identify and group barriers to healthcare access. For instance, the Barriers to Healthcare Checklist has eight domains (i.e. emotional, executive functioning, support, transportation) (Raymaker et al. 2017). The model proposed by Nicolaidis et al. does encapsulate many, but not all, of these domains (Nicolaidis et al. 2015). This conceptualisation is important to guide future research coherently, to ensure that a full account of barriers to healthcare is given, and to specify hypotheses for studies. As the research into this area is very recent (i.e. all studies are within the past 3 years) more comprehensive studies are likely to follow.

This review had several strengths. First, the agreement between the two authors who screened papers for the 10% sample of included studies (over 99%) suggests that both the selection criteria and initial screening process were valid. Second, the two authors who independently conducted the quality assessment had a very high degree of agreement. These two points suggest the papers included in the final review are likely exhaustive of the available literature and that each paper has been evaluated reliably. The search strategy was developed in an iterative way, so that search terms were refined. Experts in this research area were also included in the development of the search strategy. This study use the QATSDD to assess the quality of the included studies. This was selected a priori as a range of study methodologies was anticipated. This measure allows for a comparison between qualitative, quantitative, and mixed-methodology studies. However, it should be noted that a range of tools for different study types are available and that future studies could use multiple measures as per study type. Considering the limitations of this review, first, our review only identified a small number of papers meeting inclusion criteria. Thus, the barriers and facilitators identified in this review should not be considered definitive. It remains to be seen whether a broader set of barriers will consistently emerge when further research is conducted. Second, the exclusion criteria set for this study mean that our review may not have not have identified some facilitators of healthcare access used in clinical services. Thus, studies about clinicians’ perspectives on healthcare access for autistic people were not included. In the future, studies should explore the perspectives of autistic people, relatives/carers of autistic people, and clinicians about barriers and facilitators to healthcare access.

## Conclusions

This review identified six studies that investigated the barriers to healthcare access faced by autistic people and ways of addressing those barriers. Although the studies varied in their methodologies, three consistent findings emerged. First, autistic people find inter-personal communication with healthcare providers challenging. This can be due to literal thinking, or other communication atypicalities idiosyncratic to autistic people. However, healthcare providers may be unwilling to empower autistic people to communicate using their preferred communication style. Second, healthcare providers’ knowledge of autism may be lacking. Autistic people describe stigma and assumptions made about their abilities by healthcare providers. Yet, providers report feeling unable to provide adequate care to autistic patients, and some report a lack of formal training. Third, sensory sensitivities make accessing healthcare difficult for autistic people. This begins in the waiting/reception area, and continues through the appointment in a clinic room. Alleviating these sensitivities for autistic patients is likely to improve their healthcare access. Future research should focus on specifying a robust framework of health care barriers (for instance, operationalising what a barrier is, and creating a taxonomy of barriers), and creating tools/environments that can be used in services to reduce barriers in a way that is tailored to the person. In the UK, the NHS England 10 year plan recently announced the design and trial of an autism specific health check that will aim to improve the health care experience of autistic people (National Health Service 2019). In the US, the AASPIRE Healthcare Toolkit (www.autismandhealth.org) is being used to address barriers. If these interventions are effective, implementation across health care in a range of health settings will likely ensure improved access to usual healthcare for autistic people, and improvements in health, quality of life, and life expectancy may follow.

## Electronic Supplementary Material

Below is the link to the electronic supplementary material.

Supplementary material 1 (DOCX 13 kb)
