# Supplementary material for: A Systematic Review of What Barriers and Facilitators Prevent and Enable Physical Healthcare Services Access for Autistic Adults
Source: J Autism Dev Disord. 2019 May 23;49(8):3387–400. doi: 10.1007/s10803-019-04049-2 (PMC6647496; doi:10.1007/s10803-019-04049-2)
Supplement: Supplementary file 1 — Supplementary material 1 (DOCX 13 kb) [file 10803_2019_4049_MOESM1_ESM.docx]

**A systematic review of what barriers and facilitators prevent and enable physical healthcare services access for autistic adults**

Journal of Autism and Developmental Disorders

David Mason 1; Barry Ingham 1,2; Anna Urbanowicz 3; Cos Michael 4; Heather Birtles 5; Marc Woodbury-Smith 1,2; Toni Brown 2; Ian James 6; Clare Scarlett 7; Christina Nicolaidis 8; Jeremy Parr 1,2

1 Institute of Neuroscience, Newcastle University, UK

2 Northumberland, Tyne & Wear NHS Foundation Trust, UK

3 Global, Urban and Social Studies, RMIT University, Australia

4 Autistic advocate, UK

5 Newcastle University

6 Campus for Ageing and Vitality, Newcastle General Hospital

7 NHS North Tyneside CCG

8 Portland State University

Corresponding author: David Mason; David.Mason@newcastle.ac.uk

| Supplementary material Table S1:Decision matrix of key questions used for the title/abstract screen | | |
| --- | --- | --- |
| Inclusion: | Yes | No |
| Does the title or abstract describe a discrete sample of autistic adults? |  |  |
| Does the title or abstract give an explicit age for the participants in the study? |  |  |
| Does the title or abstract specifically describe research about barriers (features that inhibit access) to, or facilitators (features that encourage access) of, healthcare? |  |  |
| Does the title or abstract indicate the study collected primary data? (This can be quantitative, qualitative, or mixed methodology.) |  |  |
| Does the title or abstract meet the above criteria (age, facilitators, primary data) but includes a population with intellectual disabilities?* |  |  |
| Exclusion | Yes | No |
| Does the title or abstract include samples that are not comprised of autistic adults? (Nor likely to include sub-samples of autistic participants.) |  |  |
| Does the title or abstract describe research about general healthcare experiences of autistic adults, without specifying barriers or facilitators? |  |  |
| * This is acceptable as the paper may give data specifically about intellectual disability and co-occurring diagnosis of autism spectrum disorder | | |
